# The Relationship between Serum Selenium Levels and Restless Leg Syndrome in Chronic Kidney Disease Patients

**DOI:** 10.3390/medicina59101795

**Published:** 2023-10-09

**Authors:** Duygu Tutan, Jan Ulfberg, Nihal Aydemir, Barış Eser, İbrahim Doğan

**Affiliations:** 1 Department of Internal Medicine, Erol Olçok Training and Research Hospital, 19040 Çorum, Turkey; 2 Circad Health, 71330 Nora, Sweden; jan.ulfberg@circad.se; 3 Department of Nephrology, Hitit University Faculty of Medicine, 19030 Çorum, Turkey; nihalaydemir7677@gmail.com (N.A.); beser374@gmail.com (B.E.); dr.ibrahimdogan@hotmail.com (İ.D.)

**Keywords:** chronic kidney disease, selenium, restless leg syndrome

## Abstract

*Background and Objectives*: Chronic kidney disease (CKD) is a global public health issue with rising incidence linked to substantial morbidity and mortality. Selenium, an antioxidant trace element, has been linked to low serum levels in end-stage renal disease. Restless Leg Syndrome (RLS), a sleep disorder, is prevalent in CKD patients and significantly impacts their quality of life. The objective of this study was to examine the correlation between serum selenium levels and the prevalence of restless leg syndrome in individuals with chronic kidney disease. *Materials and Methods*: Forty-six CKD patients undergoing serum selenium level assessments between 1 January 2020 and 28 February 2022, at the Hitit University Faculty of Medicine Department of Nephrology Outpatient Clinic or Hemodialysis Unit, were included. Patients over 18 years of age with no history of hematological or oncological diseases or acute or chronic inflammatory conditions were included in the study groups. Patients taking selenium supplements were excluded. Demographic data, comorbidities, and laboratory values were collected, and RLS presence and severity were evaluated. Statistical analyses include descriptive statistics, correlation analysis, the Mann–Whitney U test, Student’s *t* test, and Chi-square test. *Results*: Among the 46 patients, 16 (34.78%) had RLS symptoms. The patient group included 34.78% predialysis, 34.78% peritoneal dialysis, and 30.44% hemodialysis patients, with a median age of 47.98 years. There was no difference in age, gender, and Charlson comorbidity between patients with or without RLS (*p* = 0.881, *p* = 0.702, *p* = 0.650). RLS prevalence varied across CKD subgroups, with hemodialysis patients having a higher prevalence (*p* = 0.036). Clinical parameters such as blood urea nitrogen, creatinine, calcium, phosphorus, platelet counts, and parathyroid hormone levels exhibited significant differences between patients with and without RLS (*p* < 0.05). Serum selenium levels were not significantly different between patients with and without RLS (*p* = 0.327). *Conclusions*: With an increased comorbidity burden, CKD poses a significant healthcare challenge. When accompanied by RLS, this burden can be debilitating. The difference in CKD stages between groups has shed light on a critical determinant of RLS in this population, emphasizing the role of the chronic kidney disease stage. In our study, serum selenium levels were not associated with the presence and severity of RLS. However, prospective studies with larger numbers of participants are needed to draw a definitive conclusion.

## 1. Introduction

Chronic kidney disease (CKD) is a global public health problem with increasing incidence, high morbidity, and mortality [1]. End-stage renal disease (ESRD) is the final stage of chronic kidney disease (CKD), characterized by a significant loss of kidney function. It is a severe and irreversible condition that requires renal replacement therapy (RRT) to sustain life [2]. ESRD is associated with a range of complications and comorbidities, including cardiovascular disease, depression, malnutrition, and increased mortality [2,3]. The prevalence and incidence of ESRD have been rapidly increasing worldwide, with an estimated 2.6 million patients receiving RRT in 2010, projected to rise to 5.4 million by 2030 [2].

Restless Leg Syndrome (RLS), also known as Willis–Ekbom disease, is primarily diagnosed based on a careful clinical history and a detailed physical examination. It is a feeling of discomfort, including the urge to move the legs, which increases with inactivity and rest, especially at night [4]. RLS is a taxing sleep disorder affecting approximately 5.2–15% of the general adult population [5,6]. RLS can severely affect the quality of life and day-to-day functionality and is associated with significant economic, social, and healthcare burdens [7,8]. Although it is a common disease, it is often overlooked because the diagnosis is made clinically, overlaps with other sensory and motor conditions, and is not well -known by physicians. For these reasons, it is an underdiagnosed and under-treated disease [9,10].

The pathogenesis of RLS has not been fully elucidated, but tissue ischemia due to decreased microcirculation at night, dopaminergic dysfunction, peripheral sensory neuropathy, cortical and spinal excitability, uremia-related factors, iron deficiency, anemia, and genetic and lifestyle factors are involved in the etiology [11]. It has also been reported that RLS is associated with metabolic diseases such as diabetes and insulin resistance, chronic kidney disease, inflammation, and oxidative stress [10].

RLS is more common in dialysis patients than in the normal population and is observed in 15–30% of patients [12,13]. RLS is an important condition in these patients as it impairs treatment adherence and severely affects the quality of life [14]. Studies have found that some oxidative stress markers in dialysis patients are associated with RLS in this population [15].

Oxidative stress is prominent in RLS patients, and low serum levels of selenium, an antioxidant, have been found to be related to RLS, and studies have found that there is a significant improvement in symptoms with selenium supplementation [16,17]. Selenium is a trace element with antioxidant properties. Dialysis patients are under oxidative stress, and serum selenium levels are known to be lower in these patients than in healthy populations [18].

The management of RLS over an extended period of time presents a complex and multifaceted endeavor that poses significant challenges to healthcare professionals. The protracted nature of RLS treatment necessitates a comprehensive and multifaceted approach, encompassing various therapeutic modalities and interventions aimed at mitigating distressing symptoms and improving the overall quality of life for affected individuals. The intricate nature of RLS can be alleviated by dopaminergic drugs, which are one of the primary drugs used in treatment, can increase the concentration of dopamine levels too much in the central nervous system, and worsen symptoms; this is known as augmentation. Additionally, vasodilator drugs have been tried in treatment, but their use has been abandoned due to serious side effects [19]. Gabapentinoids, oxycodone–naloxone, and various iron preparations have also been tried in treatment, but long-term effective results have not been achieved [20]. This has led to the search for new effective alternative therapies with low side effects. Studies have shown that vibration pads, cryotherapy, yoga, compression devices, acupuncture, acupressure, and hydrotherapy can improve some of the outcomes of restless leg syndrome [21,22].

When we analyzed the literature, we observed that no studies had investigated the relationship between serum selenium levels and restless leg syndrome in chronic kidney disease. The primary aim of this study was to determine whether reduced plasma selenium levels were associated with the presence and severity of restless leg syndrome.

## 2. Materials and Methods

Patients with chronic kidney disease whose serum selenium levels were evaluated between 1 January 2020 and 28 February 2022, at the Hitit University Faculty of Medicine Department of Nephrology Outpatient Clinic or Hemodialysis Unit, were selected as the subjects of the study. Seventy-five individuals over the age of 18 who did not have any known hematologic or oncologic condition, who were not malnourished, and who did not have any acute or chronic inflammatory disorders were found in the initial screening. Patients who were using selenium supplements at the time of the trial were not allowed to participate in it. The hospital archive was scanned to obtain information on patients’ ages, genders, treatment groups, and the presence of chronic illnesses such as diabetes mellitus, hypertension, coronary artery disease, congestive heart failure, chronic obstructive pulmonary disease, and peripheral artery disease. A total of 21 patients were excluded from the study due to various reasons. These reasons encompassed the unavailability of their blood results within the system, incomplete blood results, mortality within the preceding three-year period, or the inability to establish contact with the patients. In the comprehensive panel of blood tests, the following parameters were meticulously measured and documented: serum glucose, potassium, thyroid-stimulating hormone (TSH), creatinine, C-reactive peptide (CRP), platelet distribution width (PDW), uric acid, total cholesterol, hemoglobin, sodium, high-density lipoprotein (HDL), ferritin, white blood cell count (WBC), triglyceride, parathormone, monocyte, LDL (low-density lipoprotein), phosphorus levels, calcium, vitamin B12, neutrophil, lymphocyte, albumin levels, blood urea nitrogen (BUN), and serum selenium levels were also recorded. 

Hemodialysis patients had blood drawn just before the middle-of-the-week dialysis sessions, whereas predialysis and peritoneal dialysis patients had their blood drawn during their outpatient clinical controls after overnight fasting. In order to maintain consistency, the middle dialysis session of the week was selected for all patients, since they had dialysis on various days of the week. The collection of blood immediately before the beginning of the dialysis session was the preferable method in order to guarantee that the levels of serum selenium remained unaffected by the dialysis procedure. To mitigate potential impacts on lipid markers and fasting blood glucose levels, it was deemed preferable to carry out blood collection after an overnight fasting period. The chronic kidney disease epidemiology collaboration equation (CKD-EPI) was used to generate glomerular filtration rate estimates. Kt/V_urea_ was used to determine peritoneal dialysis and hemodialysis qualification. Charlson Comorbidity Scores were calculated for evaluation of the accompanying chronic diseases. Patients were contacted and invited for examination in order to analyze their RLS-related symptoms in the past 3 years. Eight patients did not want to participate in the study, stating that they did not want to come to the outpatient clinic to be evaluated for RLS. The disease severity score of patients with RLS symptoms was determined face to face according to the International Restless Legs Syndrome Study Group Diagnosis Criteria and Rating Scale [23]. This study was approved by the Hitit University Faculty of Medicine Clinical Research Ethics Committee, and a consent form was obtained from all participants who agreed to participate in the study (Decision No: 2023-79/Date: 14 June 2023).

All statistical analyses were conducted using IBM SPSS Statistics for Windows software (version 26; IBM Corp., Armonk, NY, USA). Descriptive statistics were presented as follows: categorical variables were summarized as counts and percentages, normally distributed numeric variables were expressed as mean ± standard deviation, and non-normally distributed numeric variables were presented as median (minimum, maximum) values, with the distribution of data assessed using the Shapiro–Wilk test. Correlation analysis between variables was performed using either Pearson or Spearman correlation coefficients based on the data distribution. Comparisons of numerical measurements between two independent research groups were assessed using the Mann–Whitney U test for non-normally distributed variables and the Student’s *t* test for normally distributed variables, as appropriate. The comparison of categorical variables between research groups was conducted using the Chi-square test. A level of *p* < 0.05 was considered statistically significant.

## 3. Results

Forty-six patients who met the study criteria were included in the study. There were 16 (34.78%) predialysis patients, 16 (34.78%) peritoneal dialysis patients, and 14 (30.44%) hemodialysis patients. The mean age was 47.98 years. Twenty-seven (58.7%) of the patients were male, and 19 (41.3%) were female. The median Charlson Comorbidity Index was 2 (2–9). Other characteristics of the patients are detailed in Table 1.

### Evaluation of the Variables between Groups According to Restless Leg Syndrome Groups

For the evaluation of variables between patients with restless leg syndrome and without, participants were divided into two groups. Sixteen (34.78%) patients had RLS in the previous three years. The distribution of patients among different chronic kidney disease groups revealed a significant difference (*p* = 0.036). Predialysis was more common in the No-RLS group (46.67%) compared to the RLS group (12.5%). Hemodialysis, on the other hand, was more prevalent in the RLS group (50%) compared to the No-RLS group (20%). Moreover, 57.14% of hemodialysis patients (n = 8) had RLS symptoms. There was no significant difference observed in age, gender distribution, education, and Charlson Comorbidity Index between the groups (*p* = 0.881, *p* = 0.702, *p* = 0.446, *p* = 0.650).

Several clinical parameters exhibited statistically significant differences between the two groups, including BUN, creatinine levels, calcium levels, phosphorus levels, platelet counts, and parathyroid hormone levels (*p* = 0.005, *p* = 0.005, *p* = 0.013, *p* = 0.003, *p* = 0.009, and *p* = 0.009, respectively). Other laboratory parameters, such as LDL cholesterol, uric acid, sodium, potassium, hemoglobin, neutrophil count, lymphocyte count, white blood cell count, bicarbonate, ferritin, kt/V, vitamin B12, folate, and thyroid-stimulating hormone, did not exhibit statistically significant differences between the two groups (Table 1). There was no statistically significant correlation found between serum selenium levels and restless leg syndrome in CKD patients (*p* = 0.327).

## 4. Discussion

Chronic kidney disease (CKD) poses a substantial and pressing healthcare problem owing to its progressively increasing prevalence and its intricate interplay with a myriad of comorbidities [1]. Our study encompassed 46 patients meeting stringent inclusion criteria, demonstrating an equitable distribution across three distinct chronic kidney disease stages: predialysis, peritoneal dialysis, and hemodialysis.

Selenium is an essential trace element that plays a crucial role in various physiological functions in the human body. It is involved in the regulation of metabolic and regulatory processes and serves as a cofactor for several selenoproteins involved in antioxidant defense, DNA synthesis, and immune regulation [24]. It has been hypothesized that the increased morbidity seen in HD patients may be partly due to an unrecognized trace element imbalance [25].

Oxidative stress and inflammation are integral parts of the pathophysiology of CKD and contribute to kidney damage and systemic complications [26]. Selenium deficiency, in turn, may exacerbate oxidative stress and inflammation, fostering disease progression and increasing the risk of morbidities. One of the key functions of selenium is its role as an antioxidant. Selenium-dependent enzymes, such as glutathione peroxidase, help protect cells from oxidative damage by neutralizing reactive oxygen species, and selenium also contributes to the maintenance of the redox balance in the body, preventing oxidative stress [27]. A deficiency of selenium has been unequivocally linked to a wide range of detrimental health conditions, encompassing but not limited to cardiovascular disease, neoplastic processes, and compromised immune system functionality [28]. Selenium supplementation has been found to have potential benefits in cardiovascular disease, as it can regulate the inflammatory response and protect against oxidative damage [24].

Our study evaluated the prevalence of RLS in CKD patients and its potential association with various clinical parameters. RLS, a neurologic disorder characterized by an irresistible urge to move the legs, often accompanied by uncomfortable sensations, can profoundly impact a patient’s quality of life [17]. There is limited research specifically examining the relationship between restless leg syndrome and selenium [16,29,30]. However, selenium has been studied in the context of various neurological disorders, including epilepsy, Parkinson’s disease, Alzheimer’s disease, and multiple sclerosis [31,32,33].

Chronic kidney disease is a global public health problem with increasing incidence, high morbidity, and mortality [1]. Sleep disorders and RLS are common in patients with chronic kidney disease; RLS has a prevalence of 15% to 30% in dialysis patients and patients undergoing dialysis therapy [13,34]. RLS is an important condition in these patients as it impairs adherence to treatment, has a significant impact on quality of life, and is associated with an increased risk of death [14,35,36,37]. Studies have found that some markers of oxidative stress in dialysis patients are associated with RLS in this population [15].

A noteworthy finding was the differing prevalence of RLS across the chronic kidney disease groups. Hemodialysis patients exhibited a higher prevalence of RLS compared to those in the predialysis group. Chronic kidney disease and restless leg syndrome exhibit an association; although the precise mechanisms underlying their relationship remain not entirely elucidated, both conditions commonly involve iron deficiency [34,38].

Reduced iron levels in the brain can result in abnormal dopamine activity, potentially contributing to RLS symptoms [39]. In CKD, iron deficiency may arise due to impaired absorption and diminished erythropoietin production, exacerbating RLS symptoms [39]. Several clinical parameters, including BUN, creatinine levels, calcium levels, phosphorus levels, platelet counts, serum bicarbonate, and parathyroid hormone levels, exhibited statistically significant differences between the RLS and No-RLS groups in our study, but it was noteworthy that there was no difference in ferritin levels between the groups. We attribute these disparities in clinical parameters to the various stages of CKD as opposed to a direct correlation with RLS. These results suggest that these parameters are possibly associated with the presence of RLS in renal patients, highlighting the association between CKD stage and RLS as these parameters reflect the CKD stage.

Additionally, CKD leads to the accumulation of uremic toxins in the bloodstream, affecting the nervous system and potentially triggering RLS [38]. Anemia, a commonly encountered complication in individuals with chronic kidney disease, has been documented to exhibit a correlation with the exacerbation or onset of restless leg syndrome symptoms [38], even though we did not see a difference in hemoglobin levels between our patient groups. CKD-induced peripheral neuropathy can also contribute to sensory discomfort in RLS. Various secondary factors associated with CKD, such as sleep disturbances, medications, and electrolyte imbalances, can further complicate RLS [38]. Additionally, genetic and environmental factors may predispose individuals to both CKD and RLS [40].

Studies addressing the relationship between selenium and RLS are quite rare. Studies in patients with preserved renal function have shown that selenium deficiency is associated with RLS and that selenium supplementation may reduce RLS symptoms due to possible antioxidant and partially dopaminergic effects in RLS [29]. However, contradictory to these findings, another study reported that high selenium levels may also be associated with RLS [30]. Our analysis found no statistically significant correlation between serum selenium levels and RLS in CKD patients, suggesting that it might not exert a substantial influence on RLS development or presence.

This study is significant as this is a rare study in the literature evaluating the relationship between selenium and RLS in patients with CKD. Nevertheless, this study has some limitations; the sample size in this study was relatively small, and the study was conducted at a single medical center, which may limit the generalizability of the findings. This study’s partly prospective partly retrospective design may limit the ability to establish causality.

To limit these shortcomings and strengthen our study design, we enrolled a carefully selected cohort of CKD patients, ensuring homogeneity and relevance to the research objectives. The inclusion criteria were stringent, enhancing the internal validity of the study. During the study we assessed various clinical and demographic factors, including serum selenium levels, age, comorbidity burden, and clinical parameters. This multifaceted approach tries to provide an understanding of the presence of RLS in CKD patients. The study’s identification of chronic kidney disease stages correlation with RLS in CKD patients is a significant contribution and support to the previous literature, offering and solidifying new perspectives on risk stratification and patient management in RLS.

## 5. Conclusions

In conclusion, chronic kidney disease represents a pressing healthcare challenge, marked by its escalating prevalence and association with diverse complications, including restless leg syndrome. This study, encompassing a carefully selected cohort of CKD patients, has shed light on critical determinants of RLS in this population, emphasizing the role of chronic kidney disease stage and its relationship with selenium. In our study, serum selenium levels were not associated with the presence and severity of RLS. However, prospective observational studies and randomized controlled trials with larger numbers of participants are needed to draw a definitive conclusion.

## Figures and Tables

**Table 1:** Comparison of variables between groups according to restless leg syndrome existence.

Variables		All Participants (n = 46)	No-RLS (n = 30)	RLS (n = 16)	Statistical Significance
Group	Predialysis	16 (34.78%)	14 (46.67%)	2 (12.5%)	0.036 ^‡^
	Peritoneal Dialysis	16 (34.78%)	10 (33.33%)	6 (37.5%)	
	Hemodialysis	14 (30.44%)	6 (20%)	8 (50%)	
Age		47.98 ± 11.51	48.17 ± 11.71	47.63 ± 11.51	0.881 *
Gender	Male	27 (58.7%)	17 (56.67%)	10 (62.5%)	0.702 ^‡^
	Female	19 (41.3%)	13 (43.33%)	6 (37.5%)	
Education	Primary and Middle School	32 (69.57%)	19 (63.33%)	13 (81.25%)	0.446 ^‡^
	High School	10 (21.73%)	8 (26.67%)	2 (12.5%)	
	University	4 (8.70%)	3 (10%)	1 (6.25%)	
Charlson Comorbidity Index		2 (2–9)	2 (2–9)	2 (2–5)	0.650 ^†^
Weight (kilograms)		71.44 ± 20.49	71.92 ± 21.9	70.56 ± 18.19	0.833 *
Height (meters)		1.65 ± 0.11	1.64 ± 0.12	1.66 ± 0.1	0.681 *
BMI (kg/m^2^)		24.24 (15.56–46.65)	24.06 (15.56–46.65)	25.61 (16.49–33.89)	0.908 ^†^
BUN (mg/dL)		48.3 ± 22.26	41.8 ± 18.17	60.5 ± 24.61	0.005 *
Cre (mg/dL)		6.45 (1.2–14.2)	4.65 (1.3–12.3)	10.05 (1.2–14.2)	0.005 ^†^
LDL (mg/dL)		108.2 ± 42.71	116.93 ± 45.01	91.81 ± 33.42	0.056 *
Uric Acid (mg/dL)		6.29 ± 1.56	6.41 ± 1.6	6.06 ± 1.5	0.477 *
Na (mEq/L)		139 (129–145)	140 (129–145)	138.5 (130–141)	0.338 ^†^
K (mEq/L)		4.82 ± 0.87	4.64 ± 0.65	5.15 ± 1.13	0.058 *
Ca (mg/dL)		8.82 ± 0.76	9.02 ± 0.7	8.44 ± 0.73	0.013 *
P (mg/dL)		4.33 ± 1.32	3.91 ± 0.94	5.1 ± 1.59	0.003 *
Hb (mg/dL)		11.33 ± 1.68	11.4 ± 1.9	11.2 ± 1.21	0.700 *
Neu (10^9^/L)		4.3 (2.17–10)	4.03 (2.47–10)	4.8 (2.17–7.92)	0.189 ^†^
Lym (10^9^/L)		1.75 ± 0.6	1.77 ± 0.66	1.72 ± 0.49	0.784 *
WBC (10^9^/L)		7.14 ± 1.87	6.96 ± 1.95	7.48 ± 1.72	0.372 *
Plt (10^9^/L)		220 (42–480)	197 (42–390)	233 (160–480)	0.009 ^†^
PTH (pg/mL)		275 (15–1660)	205.5 (15–989)	398.5 (38–1660)	0.009 ^†^
HCO3 (mmol/L)		23.23 ± 3.49	24.21 ± 2.88	21.39 ± 3.87	0.008 *
Ferritin (ng/mL)		273.5 (11–2000)	278 (11–2000)	273.5 (38–1052)	0.872 ^†^
kt/V		1.9 (0.97–4.93)	2.08 (1.4–3.45)	1.72 (0.97–4.93)	0.070 ^†^
Vitamin B12 (pg/mL)		420 (140–2000)	390 (140–2000)	460 (240–1040)	0.406 ^†^
Folate (ng/mL)		9.2 (3.5–20)	9.2 (3.5–20)	9.5 (4.2–20)	0.215 ^†^
TSH (mIU/L)		1.65 (0.4–5.7)	1.6 (0.5–5.7)	2.33 (0.4–5.1)	0.070 ^†^
Selenium (ng/mL)		67.3 (46.1–121.8)	68 (46.1–92.5)	65.5 (46.1–121.8)	0.327 ^†^
RLS Severity Score				14.94 ± 8.64	
RLS Severity	Mild			6 (37.5%)	
	Moderate			6 (37.5%)	
	Severe			3 (18.75%)	
	Very Severe			1 (6.25%)	

BMI: Body mass index, BUN: Blood urea nitrogen, Cre: Creatinine, LDL: Low-density lipoprotein, Na: Sodium, K: Potassium, Ca: Calcium, P: Phosphorus, Hb: Hemoglobin, Neu: Neutrophil count, Lym: Lymphocyte count, WBC: White blood cell count, Plt: Platelet count, PTH: parathormone, HCO3: Bicarbonate, TSH: thyroid-stimulating hormone, RLS: restless leg syndrome, * Student’s *t* test results, ^†^ Mann–Whitney U test results, ^‡^ Chi-square test results.

## Data Availability

The data presented in this study are available on request from the corresponding author. The data are not publicly available due to privacy.
